# An Evaluation of 3-Rhamnosylquercetin, a Glycosylated Form of Quercetin, against the Myotoxic and Edematogenic Effects of **sPLA**_**2**_ from *Crotalus durissus terrificus*

**DOI:** 10.1155/2014/341270

**Published:** 2014-02-18

**Authors:** Daniela de Oliveira Toyama, Henrique Hessel Gaeta, Marcus Vinícius Terashima de Pinho, Marcelo José Pena Ferreira, Paulete Romoff, Fábio Filippi Matioli, Angelo José Magro, Marcos Roberto de Mattos Fontes, Marcos Hikari Toyama

**Affiliations:** ^1^Centro de Ciências Biológicas e da Saúde (CCBS), Universidade Presbiteriana Mackenzie, 01302-907 São Paulo, SP, Brazil; ^2^Campus Experimental do Litoral Paulista, UNESP, Laboratório de Biologia Molecular e Peptídeos, BIOMOLPEP, 11330-900 São Vicente, SP, Brazil; ^3^Programa de Pós-Graduação em Farmacologia, Faculdade de Ciências Médicas, UNICAMP, 13083-970 Campinas, SP, Brazil; ^4^Escola de Engenharia, Universidade Presbiteriana Mackenzie, 01302-907 São Paulo, SP, Brazil; ^5^Departamento de Física e Biofísica, Instituto de Biociências, UNESP, 18618-970 Botucatu, SP, Brazil

## Abstract

This paper shows the results of quercitrin effects on the structure and biological activity of secretory phospholipase (sPLA_2_) from *Crotalus durissus terrificus*, which is the main toxin involved in the pharmacological effects of this snake venom. According to our mass spectrometry and circular dichroism results, quercetin was able to promote a chemical modification of some amino acid residues and modify the secondary structure of *C. d. terrificus* sPLA_2_. Moreover, molecular docking studies showed that quercitrin can establish chemical interactions with some of the crucial amino acid residues involved in the enzymatic activity of the sPLA_2_, indicating that this flavonoid could also physically impair substrate molecule access to the catalytic site of the toxin. Additionally, *in vitro* and *in vivo* assays showed that the quercitrin strongly diminished the catalytic activity of the protein, altered its Vmax and Km values, and presented a more potent inhibition of essential pharmacological activities in the *C. d. terrificus* sPLA_2_, such as its myotoxicity and edematogenic effect, in comparison to quercetin. Thus, we concluded that the rhamnose group found in quercitrin is most likely essential to the antivenom activities of this flavonoid against *C. d. terrificus* sPLA_2_.

## 1. Introduction

At present, phospholipase A_2_s (PLA_2_s) (EC 3.1.1.4) can be classified into various groups and subgroups according to a complex molecular taxonomy. Several PLA_2_s have recently been isolated and characterized. One of the most investigated groups of PLA_2_s includes the secretory phospholipase A_2_s (sPLA_2_), which are primarily found in the venom of several animals. The sPLA_2_s exhibit well-established functions in the digestion of dietary phospholipids, although they also have important functions in the host's defense against bacterial infections, and they are involved in pathological processes such as atherosclerosis and cancer [1, 2]. Moreover, mammalian genomes encode several types of sPLA_2_-binding proteins, indicating that sPLA_2_s may have enzyme-independent activities related to their ability to bind to cellular target proteins [3]. Several recent studies have shown that snake venom sPLA_2_s present a mechanism of action that is very similar to that of human sPLA_2_s [4, 5], and some secretory phospholipase A_2_s purified from humans can induce pharmacological events similar to those of snake venom phospholipase A_2_ [6].

Thus, there is great interest in using snake venom sPLA_2_s as molecular target model to evaluate and investigate for natural compounds that potentially inhibit the activities of phospholipase A_2_ homologous molecules in other organisms [7–9]. This approach could be especially useful for developing better comprehension of several inflammatory diseases, considering the role of sPLA_2_s in the acute inflammation process and the fact that their uncontrolled production can contribute to the exacerbation of these pathological processes [10–12]. In this regard, the search for new molecules capable of significantly reducing the enzymatic activity of sPLA_2_ and decreasing the production of arachidonic acid through this route is very important from a therapeutic standpoint [13, 14].

Various natural compounds have the potential to inhibit or negatively modulate the activities of PLA_2_s and other enzymes involved in the cascade of arachidonic acid, consequently presenting a potential method for reducing and controlling the inflammatory process. The compounds known as flavonoids present remarkable anti-inflammatory activity; these molecules can inhibit the enzymatic activity of PLA_2_s and other enzymes involved in the arachidonic acid pathway, and they can reduce the synthesis of some inflammatory intermediates [15–17]. The most common natural flavonoid is quercetin (Q), which is generally found in its glycosylated forms as quercitrin (Qn) or rutin (quercetin rutinoside).

Although some studies indicate that Q has a more pronounced effect in downregulating the inflammatory response relative to Qn, other studies highlight a significant anti-inflammatory effect from both glycosides (Qn and rutinoside) in experimental colitis models in rats and other biological essays [17, 18]. Thus, the main objective of this work is to clarify, from a structural point of view, the effects of quercitrin's anti-inflammatory properties and the influence of its structural properties on the edema and myonecrosis induced by sPLA_2_ purified from *C. d. terrificus*.

In this study, we performed experimental and theoretical procedures including chromatography, circular dichroism, molecular docking, and other *in vitro* and *in vivo* biological essays to evaluate the effects of Q and Qn on *C. d. terrificus* sPLA_2_. The results obtained from these experiments showed that Qn is a more effective inhibitor of important *C. d. terrificus* sPLA_2_ biochemical and pharmacological activities than Q, indicating that the deoxy sugar rhamnose group is most likely involved in the anti-inflammatory and antimyotoxic properties presented by the glycosylated molecular form of quercetin (Q).

## 2. Material and Methods

### 2.1. Materials

The venom from *Crotalus durissus terrificus* (*C. d. terrificus*) was kindly donated by the Butantan Institute (São Paulo, Brazil). The solvents, chemicals, and reagents used for protein purification and characterization (HPLC grade or higher) were acquired from Sigma-Aldrich Chemicals (3050 Spruce St., St. Louis, MO 63103, USA), Merck (One Merck Drive, Whitehouse Station, NJ, USA), and Bio-Rad (USA). Male Swiss mice (20–25 g) were obtained from the Multidisciplinary Center for Biological Research (CEMIB) of the State University of Campinas (UNICAMP). The animals were maintained under standard conditions (22 ± 2°C; 12 h light/dark cycle), with food and water available *ad libitum*. All animal experiments were performed in accordance with Brazilian laws for the Care and Use of Laboratory Animals, and the protocols were approved by the Committee of Ethics from UNICAMP number 2898-1.

### 2.2. Purification of Quercitrin

Quercitrin (Qn) was purified from the leaves of *Baccharis microdonta* DC. that were collected in Campos do Jordão, SP, in June 2008. A voucher specimen has been deposited at the Herbarium of Prefeitura Municipal de São Paulo (PMSP) under number 8980. Dried and powdered leaves (241 g) were defatted with n-hexane and subsequently extracted with methanol at room temperature. Following its concentration under a vacuum, the crude MeOH extract (97.5 g) was suspended in MeOH : H_2_O (1 : 1), and successively partitioned with hexanes (6.55 g), CH_2_Cl_2_ (6.69 g), EtOAc (11.83 g) and n-BuOH (18.87 g). Part of the EtOAc phase (8.0 g) was dissolved with hot methanol, resulting in a precipitate (1.70 g) and a soluble fraction (6.11 g). The soluble portion was then subjected to gel filtration on Sephadex LH-20 eluted with MeOH to make 10 fractions (A1–A10). Fraction A5 (108.8 mg) was subjected to HPLC purification to obtain the Qn flavonoid, which was identified on the basis of its UV, ESI-MS, and NMR data in comparison with data reported in the literature [19].

### 2.3. Purification of Phospholipase A_2_

To purify the *C. d. terrificus* sPLA_2_, whole venom was first fractionated as previously described by [20]. Dried venom (45 mg) was dissolved in ammonium bicarbonate buffer (0.2 M, pH 8.0) and clarified by centrifugation (4,500 ×g, 1 min). The supernatant was injected into a molecular exclusion HPLC column (Superdex 75,1 × 60 cm, Pharmacia), and the chromatographic run was performed with a flow rate of 0.2 mL/min for the elution of fractions. The absorbance was monitored at 280 nm. The separated crotoxin-like fraction was immediately lyophilized. The lyophilized fraction was then subjected to reverse-phase chromatography using a *μ*-Bondapak C18 column (0.39 × 30 cm) with a flow rate of 1 mL/min for fraction elution. The absorbance was monitored at 280 nm. Afterwards, this fraction was eluted by using a nonlinear gradient with buffer A (0.1% of trifluoroacetic acid in Milli-Q water) and buffer B (acetonitrile 66% in buffer A). The final fraction was the *C. d. terrificus* sPLA_2_, and its purity was evaluated by tricine SDS-PAGE and mass spectrometry on a MALDI-TOF mass spectrometer as previously described by [21].

### 2.4. Treating sPLA_2_ with Quercetin and Quercitrin

The incubations of *C. d. terrificus* sPLA_2_ with purified quercetin (Q) and quercitrin (Qn) at (mol : mol) were performed according to the procedure described by [21]. Q and Qn were dissolved in dimethyl sulfoxide (DMSO). The concentration of DMSO never exceeded 1% during incubation. Q or Qn (400 *μ*L of 0.1 mM solution) was added to 400 *μ*L of a homogenized, purified *C. d. terrificus* sPLA_2_ solution (1 mg/mL). The mixture was then incubated for 90 min at room temperature, and 200 *μ*L aliquots were loaded into a preparative reverse-phase column to separate the treated enzyme (sPLA_2_ : Q and sPLA_2_ : Qn). Following column equilibration with HPLC buffer A (aqueous 0.1% TFA), the samples were eluted by using a discontinuous gradient of HPLC buffer B (66.6% of acetonitrile in 0.1% TFA) at a constant flow rate of 1.0 mL/min. The chromatographic run was monitored at 214 nm.

### 2.5. Circular Dichroism Spectroscopy

The secondary structure can be determined by CD spectroscopy in the “far-UV” spectral region (190–250 nm). At these wavelengths the chromophore is the peptide bond, and the signal arises when it is located in a regular, folded environment. The CD spectrum of a protein in the “near-UV” spectral region (250–350 nm) is sensitive to certain aspects of tertiary structure of proteins. At these wavelengths the chromophores are the aromatic amino acids and disulfide bonds, and the CD signals they produce are sensitive to the overall tertiary structure of the protein.

In this study, we used both assay types to evaluate the secondary structure and monitor shifts in the tertiary structure of native sPLA_2_ and sPLA_2_s that were chemically modified by quercitrin. To determine the protein secondary structure, sPLA_2_, sPLA_2_ : Q, and sPLA_2_ : Qn were dissolved in 10 mM sodium phosphate buffer (pH 7.4), and the final protein concentrations were adjusted to 8.7 mM. This protein solution was then subjected to centrifugation at 4,000 ×g for 5 min, and the resulting supernatant was transferred to a 1 mm path-length quartz cuvette. Circular dichroism spectra within a wavelength range of 185–300 nm were acquired in-house with a J720 spectropolarimeter (Jasco Corp., Japan) by using a bandwidth of 1 nm and a response time of 1 s. Data collection was performed at room temperature with a scanning speed of 100 nm/min. Nine scans were obtained for each sample, and all spectra were corrected by subtracting buffer blanks. The near-UV CD spectrum (>250 nm) of the samples provided information on the tertiary protein structure. The signals obtained in the range of 250–300 nm were caused by the absorption, dipole orientation, and the nature of the surrounding environment around the phenylalanine, tyrosine, cysteine (or S-S disulfide bridges), and tryptophan residues in the protein. In this study, the CD HPLC detector from Jasco Corp., Japan, was used to enable the scanning of sPLA_2_, sPLA_2_ : Q, and sPLA_2_ : Qn peaks.

### 2.6. Molecular Docking

For quercitrin (Qn) in silico design and docking simulations, the Avogadro v.0.9.4 (http://avogadro.openmolecules.net/) program was used to generate the *in silico* model and improve its overall structure through a steepest-descent algorithm for energy minimization based on the MMF94 force field. All docking simulations between the Qn model and the *C. d. terrificus* sPLA_2_ crystallographic structure (PDB ID 2QOG) [22] were executed with the GOLD v.5.0.1 (CCDC Software Limited, Cambridge, UK) program [23]. The docking site was defined within a 10 Å radius around the His48 residue located at the catalytic site of monomers A and C of the *C. d. terrificus* sPLA_2_ crystallographic structure. Additionally, other cavities on the protein surface were tested to identify other potential docking sites. The N*δ*1 atoms from the catalytic histidine of the *C. d. terrificus* sPLA_2_ crystallographic model were protonated, and the simulations generated approximately 1000 docking solutions to provide a representative population. The remaining docking parameters were defined according to the GOLD v.5.0.1 default settings. The docking solutions between Qn and the *C. d. terrificus* sPLA_2_ structural model were scored and rescored by using the GoldScore fitness function and the number of H-bonds between the protein and the Qn, respectively. The GoldScore fitness is the sum of the protein-ligand bond energy, protein-ligand van der Waals (vdw) energy, ligand internal vdw energy, ligand torsional strain energy, and the ligand intramolecular hydrogen bond energy. This sum represents the amount of docked complex, but it can be excessively high in the case of weak bonds. Therefore, the number of H-bonds between the protein and the Qn was also observed when choosing the best docking solutions [23].

### 2.7. Enzymatic Assay of sPLA_2_

sPLA_2_ activity was measured by following the protocols described in [24] for a 96-well plate assay using 4-nitro-3-octanoyloxy-benzoic acid (NABA or NOB, manufactured by BIOMOL, USA) as the substrate. Enzyme activity, which was expressed as the initial velocity of the reaction (Vo), was calculated on the basis of the increase in absorbance after 20 min. All assays were performed by using *n* = 12 and the absorbance at 425 nm was measured by using a SpectraMax 340 multiwell plate reader (Molecular Devices, Sunnyvale, CA). After the addition of sPLA_2_ (20 *μ*g), the reaction mixture was incubated for 40 min at 37°C, and the absorbance was read at 10 min intervals. The effect of the substrate concentration on enzyme activity was determined by measuring the absorbance increase after a 20 min incubation in Tris-HCl buffer, pH 8.0, at 37°C. All assays were performed in triplicate, and the absorbance at 425 nm was measured by using a SpectraMax 340 multiwell plate reader (Molecular Devices, Sunnyvale, CA). The remaining enzymatic assay was conducted as described above. Q and Qn were dissolved in 1% DMSO.

### 2.8. Paw Edema

A paw edema assay was performed by using the protocol described by [21]. Male Swiss mice (21 g) were anaesthetized by inhaling halothane. Posterior paw edema was induced by a single subplantar injection of sPLA_2_ that was previously incubated with samples of native sPLA_2_ and sPLA_2_ and previously treated with both flavonoids (10 *μ*g per paw). The paw volumes were measured immediately before the injection and at selected time intervals thereafter (0, 30, 60, 120, and 240 minutes) by using a hydroplethysmometer (model 7150, Ugo Basile, Italy). All drugs were dissolved in 0.9% sterile saline solution. The results are expressed as the increase in paw volume (mL) calculated by subtracting the initial volume. The area under the time-course curve was also calculated (trapezoidal rule), and the results were expressed as the total edema volume (milliliters per paw).

### 2.9. Evaluation of Myonecrosis

The liberation of creatine kinase (CK) from damaged muscle cells was determined by recording the enzyme activity in mouse plasma by using the CK-NAc kit (http://www.laborlab.com.br/, Laborlab, Brazil) as described in [25]. Native sPLA_2_ and sPLA_2_ were previously treated with both Q and Qn. These samples were injected into the left gastrocnemius muscle of male Swiss mice (18–20 g; *n* = 5). The right gastrocnemius muscle was injected with 50 *μ*L of 0.5 mg/mL sPLA_2_ samples. Control mice received an equal volume of 0.15 M NaCl. After 3 h, the mice were anesthetized, and blood was collected from the abdominal *vena cava* into tubes containing heparin as an anticoagulant. The plasma was stored at 4°C for a maximum of 12 h before the assay. The amount of CK was then determined with 40 *μ*L of plasma, which was incubated for 3 minutes at 37°C with 1.0 mL of the reagent according to the kit protocol. The resulting activity was expressed in U/L.

### 2.10. Statistical Analysis

Results are reported as the means ± SEM of replicated experiments. The significance of differences between means was assessed by an analysis of variance followed by Dunnett's test when several experimental groups were compared to the control group. The confidence limit for significance was 5%.

## 3. Results

### 3.1. Purification of Chemically Treated sPLA_2_ and sPLA_2_

**Figure 1:**
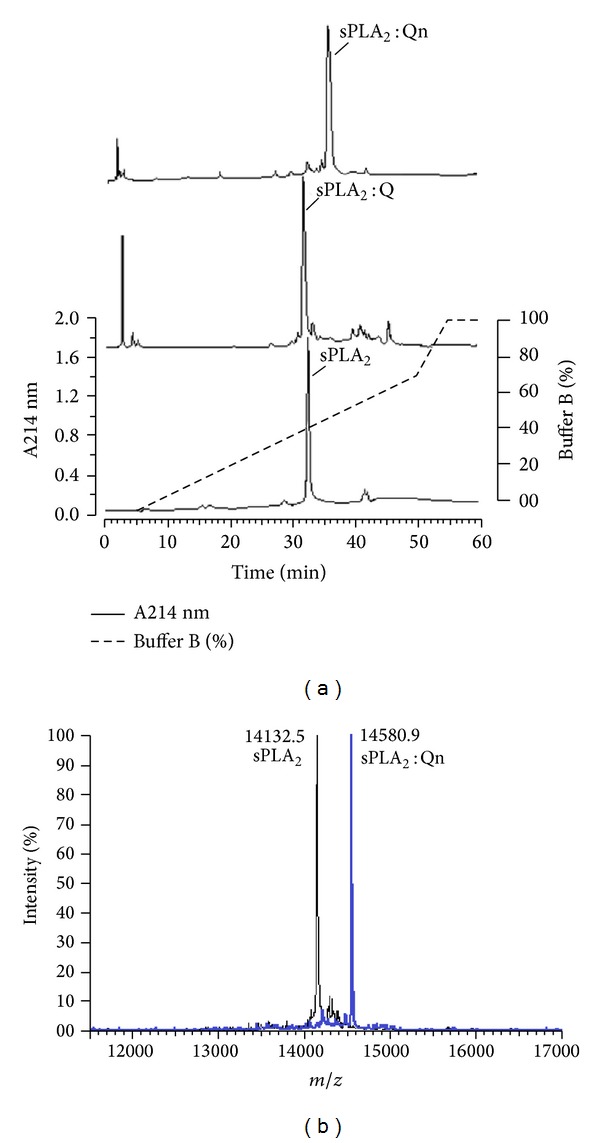
Purification and chemical modification of secretory phospholipase A_2_ (sPLA_2_). A fractionation of the whole venom was performed by reverse-phase HPLC (C5 column, 0.10 cm × 25 cm) using a nonlinear concentration gradient of buffer to obtain a high-purity protein. (a) shows a comparative profile of native sPLA_2_, sPLA_2_ : Q, and sPLA_2_ : Qn when subjected to reverse-phase HPLC. (b) shows the MALDI-TOF mass spectrometry analysis of native sPLA_2_ and sPLA_2_ : Qn, indicating the difference in the molecular mass corresponding to one molecule of bound quercitrin.

Figure 1(a) shows the chromatography profiles of the eluted native sPLA_2_, sPLA_2_ : Q, and sPLA_2_ : Qn. The retention times of native sPLA_2_, sPLA_2_ : Q, and sPLA_2_ : Qn were 32.5, 31.8, and 33.5 minutes, respectively. All sPLA_2_, sPLA_2_ : Q, and sPLA_2_ : Qn samples were lyophilized and stored for future analysis.  Figure 1(b) shows the mass spectrometry profile of native sPLA_2_ and sPLA_2_ : Q, which was the same as that found by [21]; this finding shows that the methods used here were stable, and they generated reliable and accurate data. Furthermore, the analysis result of sPLA_2_ mass spectrometry: Qn was 14580.90, so this mass is the product of sPLA_2_ incubation with Qn. Thus, Figure 1(b) only shows the results of sPLA_2_: Qn, and the results of the incubation of the product of sPLA_2_: Q were presented in the Figure 1(b).

### 3.2. Circular Dichroism Analysis

**Figure 2:**
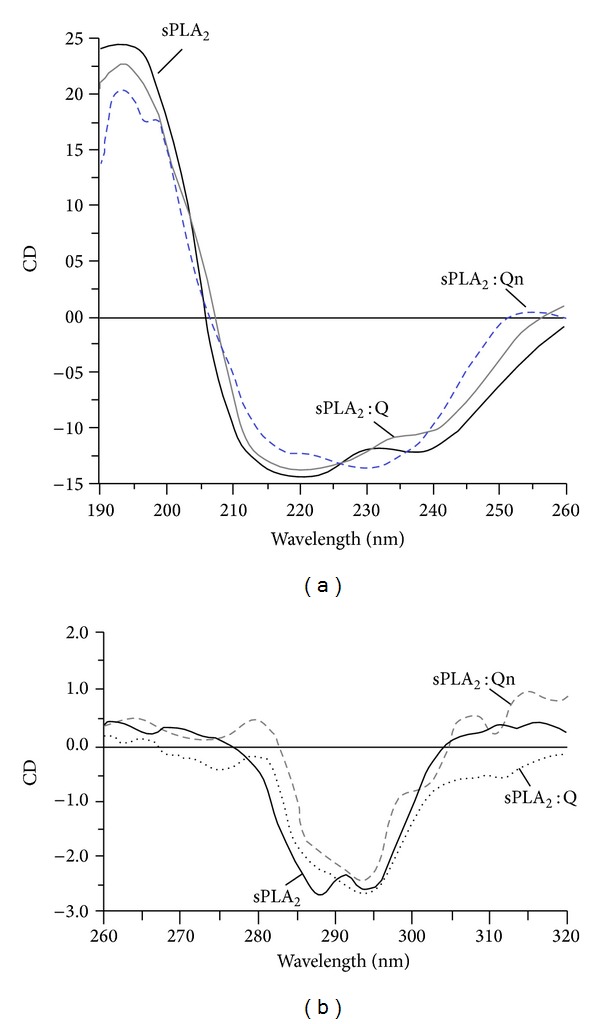
The far-UV (ultraviolet) CD spectrum of proteins can reveal important characteristics of their secondary structure. (a) shows the results of CD spectra from native sPLA_2_, sPLA_2_ : Q, and sPLA_2_ : Qn. Data from 185–280 nm are shown. The CD spectra are expressed in theta machine units in millidegrees. The near-UV CD spectrum (>250 nm) of proteins provides information on the tertiary structure. The signals obtained in the 250–300 nm region are caused by the absorption, dipole orientation, and the nature of the surrounding environment around the phenylalanine, tyrosine, cysteine (or S-S disulfide bridges), and tryptophan amino acids. (b) shows the near-UV CD spectrum of the native sPLA_2_, sPLA_2_ : Q, and sPLA_2_ : Qn.

Figure 2 shows the circular dichroism profile of the native sPLA_2_, sPLA_2_ : Qn, and sPLA_2_ : Q, which were subjected to the same test conditions. The far-UV region (ultraviolet) ranging between 190 and 260 nm was used to reveal important features of its secondary structure. The results are shown in Figure 2(a), indicating that Qn was able to induce some secondary modifications in native sPLA_2_ in comparison with quercetin (Q). In addition, Qn was able to induce a significant change in the random coil region of native sPLA_2_. The near-UV CD spectrum (>250 nm) of protein provides information on the tertiary structure. The signals obtained in the 250–300 nm region are caused by absorption, dipole orientation, and the nature of the environment surrounding the phenylalanine, tyrosine, cysteine (or S-S disulfide bridges), and tryptophan amino acids.  Figure 2(b) shows the UV CD spectrum of native sPLA_2_, sPLA_2_ : Q, and sPLA_2_ : Qn, and from these results, the previous native sPLA_2_ treatment with Qn induced more evident tertiary shifts than native sPLA_2_.

### 3.3. Molecular Docking of sPLA_2_ with Compounds

**Figure 3:**
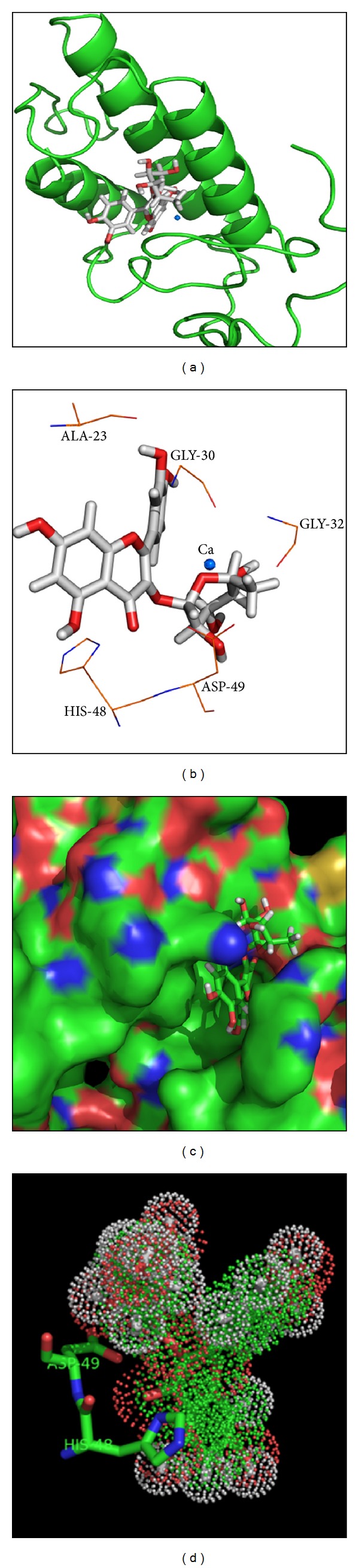
Structural representation of Q bound to sPLA_2_ from docking simulations. (a) shows a cartoon representation of the sPLA_2_ structure. quercetin is shown in a stick representation, and the Ca^2+^ ion is represented as a blue sphere. (b) shows the quercetin molecule and its main amino acid interactions. (c) shows a surface representation of sPLA_2_ bound to a quercetin molecule (stick representation). (d) shows the dot representation of the Quercetin molecule in the bound position. Two amino acid residues (Asp49 and His48) from sPLA_2_ are represented as sticks.

In addition to chromatographic and biophysical experiments, docking studies were also performed with the Cro crystallographic model [22], and they were used to analyze the probable preferential orientation of the ligands (Q and Qn) in a complex with the *C. d. terrificus* sPLA_2_. Based on the docking scores, this computational analysis showed that Qn has a higher affinity for the active site of *C. d. terrificus* sPLA_2_ than Q. The Avogadro v.0.9.4 (http://avogadro.openmolecules.net/) program was used to generate an in silico model of Qn and improve its overall structure through a steepest-descent algorithm for energy minimization based on the MMF94 force field. All docking simulations between the Qn model and the *C. d. terrificus* sPLA_2_ crystallographic structure (PDB ID 2QOG) [22] were performed with the GOLD v.5.0.1 (CCDC Software Limited, Cambridge, UK) program [23]. The docking site was defined by a 10 Å radius around the His48 residue, which was located at the catalytic site of the A and C monomers of the *C. d. terrificus* sPLA_2_ crystallographic structure. Additionally, other cavities on the protein surface were also tested to identify other potential docking sites. The N*δ*1 atoms from the catalytic histidine in the *C. d. terrificus* sPLA_2_ crystallographic model were protonated, and the simulations generated approximately 1000 docking solutions to provide a representative population. The remaining docking parameters were defined according to the GOLD v.5.0.1 default settings. The docking solutions between Qn and the *C. d. terrificus* sPLA_2_ structural model were scored and rescored by using the GoldScore fitness function. As shown in Figure 3, the main interactions between the ligand and the protein involve amino acid residues Asp49, His48, and Gly30 and the Ca^2+^ ion.

### 3.4. Enzymatic Assays

**Figure 4:**
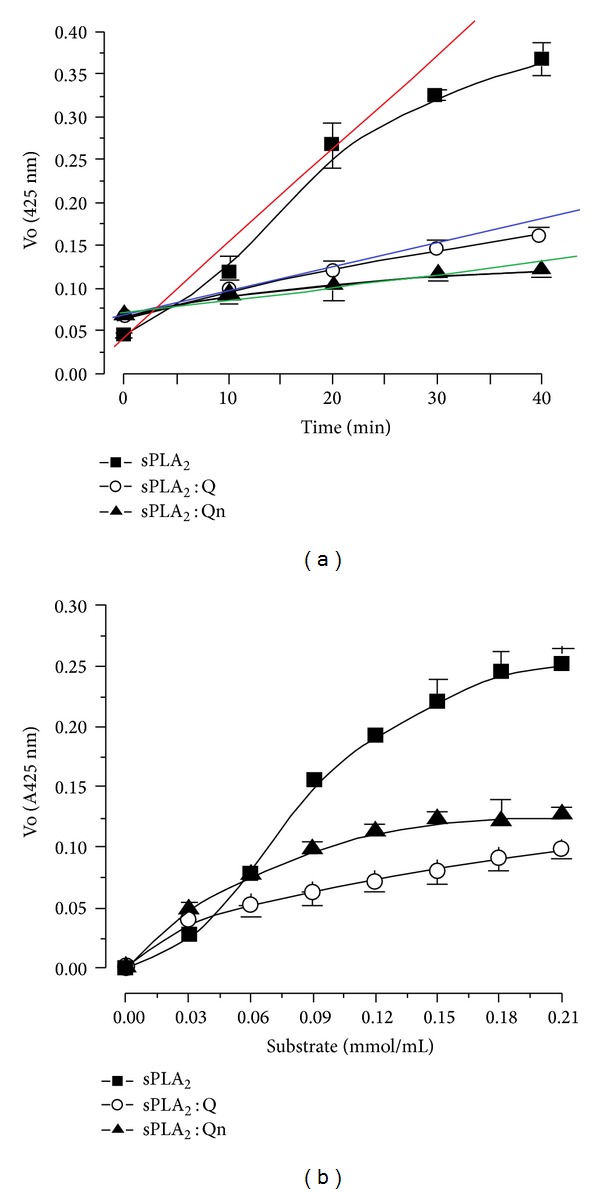
(a) shows the results of the enzymatic activity assays that were performed by using a synthetic chromogenic substrate for PLA_2_ (NOBA). The reaction was monitored at 425 nm. sPLA_2_ : Q and sPLA_2_ : Qn exhibited a significant decrease in activity when compared to native sPLA_2_. (b) shows the effect of the substrate concentration on enzyme activity in the presence of native sPLA_2_, sPLA_2_ : Q, and sPLA_2_ : Qn.

All enzymatic assays yield a product that is linear over a short period of time at an initial rate after the beginning of the enzyme activity (when performed under appropriate conditions). The linear slope indicates that the rate of the enzymatic reaction and the increase in product formation are proportional to the enzyme reaction. As the reaction proceeds, the substrate is consumed and the acceleration decreases.  Figure 4(a) shows the time-course effect of an enzymatic reaction. The native sPLA_2_ exhibited a linear rate increase over a 20 min reaction and the sPLA_2_ : Q and sPLA_2_ : Qn experienced a reduction in enzymatic activity of approximately 57 ± 4% and 63 ± 12%, respectively, in the same time period (Figure 4(a)). In fact, there is no statistically significant difference in Figure 4(a) between both inhibitors at 20 minutes of enzyme kinetic experiments. The data in Figure 4(a) suggest a trend towards greater Qn inhibition over Q. The results of Figure 4(a) show that the saturation of the active site of sPLA_2_ in the presence of Qn already occurs after 40 minutes whereas the sPLA_2_ incubated with Q, the active site of sPLA_2_ is saturated after 30 minutes. These results suggest that the inhibition profile of Q to Qn is different and that these compounds have slightly different inhibition capabilities of sPLA_2_ when it is purified from the *Crotalus durissus terrificus* venom, and the inhibitions induced by Q or Qn were statistically similar. The sPLA_2_ of *Crotalus durissus terrificus* has been characterized as an allosteric enzyme in the presence of 4-nitro-3-(octanoyloxy)benzoic acid (NOBA or NOB), which is a chromogenic substrate specific for phospholipase A_2_ [25–27].  Figure 4(b) shows the substrate effects on the sPLA_2_ activity, and the native sPLA_2_ exhibited a Vmax value of 0.254 ± 0.09 and a Km value of 0.08 ± 0.002, whereas sPLA_2_ : Qn and sPLA_2_ : Q had Vmax values of 0.12 ± 0.03 and 0.10 ± 0.03, and Km values of 0.04 ± 0.002 and 0.051 ± 0.004, respectively.

### 3.5. Pharmacological Assays

**Figure 5:**
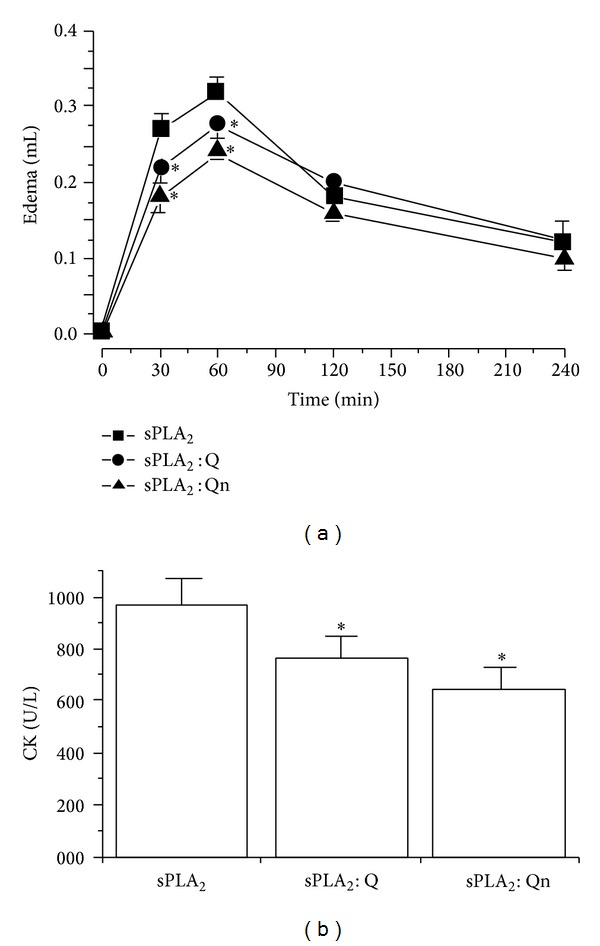
(a) shows the results of paw edema that was induced after the injection of sPLA_2_, sPLA_2_ : Q, and sPLA_2_ : Qn into the right paws of Swiss mice. Measurements were made after 30, 60, 120, and 240 min, and all the edema results expressed in (a) were obtained by subtracting the saline injection values. (b) shows the myonecrosis levels as evaluated by CK levels in Swiss mice. Fifty micrograms of native sPLA_2_, sPLA_2_ : Q, and sPLA_2_ : Qn at a final concentration of 0.5 mg/mL were injected into the gastrocnemius muscle. The results are expressed as units of enzymatic activity per liter (U/L). Error bars indicate the SEM. *P < 0.05 compared to native sPLA_2_.

The native sPLA_2_ had a maximum edema value of approximately 30 to 60 min, with a swelling value of 0.27 ± 0.06 mL (*n* = 5, and *P < 0.05) and 0.32 ± 0.04 mL (*n* = 5, and *P < 0.05) for this time interval. Within the same time interval, sPLA_2_ : Q showed maximum edema of 0.18 ± 0.04 mL (*n* = 5, *P < 0.05) and 0.28 ± 0.05 mL (*n* = 5, *P < 0.05) in the same time interval. Furthermore, sPLA_2_ : Qn showed maximum edema values of 0.18 ± 0.05 mL (*n* = 5, *P < 0.05) and 0.023 ± 0.05 mL (*n* = 5, *P < 0.05), respectively. These results showed that both Q and Qn significantly inhibit sPLA_2_ enzyme activity, and the inhibition by Qn was two times higher than that of Q (Figure 5(a)).  Figure 5(b) shows the myotoxic activity induced by native sPLA_2_, sPLA_2_ : Q, and sPLA_2_ : Qn. The extent of the damage caused by sPLA_2_ to skeletal muscles was assessed by quantifying the CK levels, which are widely used as an indirect marker of muscle damage. For trials with snake toxins, CK is used as a marker to assess the damage to skeletal muscles in the presence of snake venom. Three hours after the native sPLA_2_ injection, the CK value was 1,230 ± 270 U/L (*n* = 5, *P < 0.05). For the sPLA_2_ : Q and sPLA_2_ : Qn, the serum CK levels were 780 ± 120 U/L (*n* = 5, *P < 0.05) and 680 ± 69 (*n* = 5, *P < 0.05), respectively.

**Figure 6:**
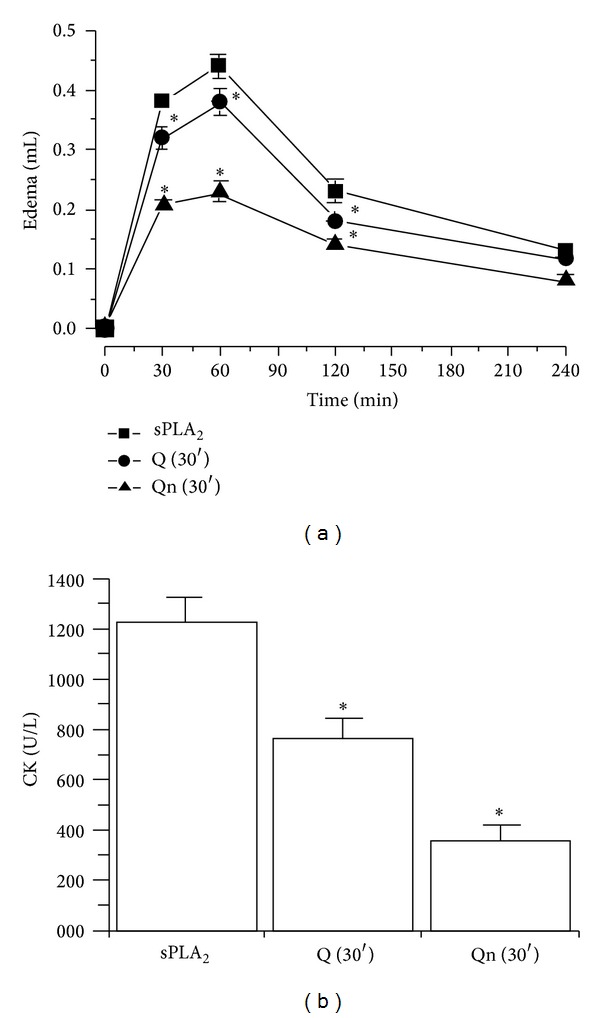
(a) shows the results from paw edema in the animals that were injected with quercitrin (Qn 30′) and quercetin (Q 30′) 30 min before sPLA_2_ administration into the right paw of Swiss mice. The control group received a saline injection prior to the administration of sPLA_2_. Measurements were made after 30, 60, 120, and 240 min, and all edema results expressed in (a) were obtained after subtracting the edema values from the saline injection. (b) shows the results of paw edema in animals that were injected with quercitrin (Qn 30′) or quercetin (Q 30′) 30 min before the administration of sPLA_2_. The control group received saline. Myonecrosis was evaluated on the basis of CK levels after 50 mg of native sPLA_2_ was injected at a final concentration of 0.5 mg/mL into the gastrocnemius muscle. The results are expressed as units of enzymatic activity per liter (U/L). Error bars indicate the SEM. *P < 0.05 compared to native sPLA_2_.

In addition to trials with sPLA_2_s that had been chemically treated with both flavonoids, assays in which the animals were pretreated with 100 *μ*L (0.3 mM/mL, IP injection, *n* = 5 and *P < 0.05) of Q and Qn were also performed.  Figure 6(a) shows the edema of animals pretreated with both flavonoids. The results of the edema assay for animals receiving 0.9% saline (100 *μ*L, IP injection, *n* = 5 and *P < 0.05) were 0.38 ± 0.06 mL (*n* = 5 and *P < 0.05) and 0.44 ± 0.05 mL (*n* = 5 and *P < 0.05) at 30 min and 60 min into the edema time-course experiment after the injection of native sPLA_2_, respectively. The animals that received Q (30′) had 0.28 ± 0.03 mL edema values at 30 min (*n* = 5, *P < 0.05) and 0.38 ± 0.08 mL at 60 min (*n* = 5, *P < 0.05). The animals treated with Qn (30′) had swelling times of 0.21 ± 0.04 mL at 30 min (*n* = 5, *P < 0.05) and 0.23 ± 0.09 mL at 60 min (*n* = 5, *P < 0.05).

Figure 6(b) shows the effects of injecting Q and Qn into animals 30 min before sPLA_2_ administration, which were injected into the left gastrocnemius muscle of male Swiss mice. The group that received saline (control group) exhibited CK levels of 970 ± 156 U/L (100 *μ*L, IP injection, *n* = 5,  *P < 0.05). The group that received Q and Qn showed a plasma CK level of 870 ± 96 U/L (100 *μ*L, IP injection, *n* = 5, *P < 0.05) and 380 ± 122 U/L (100 *μ*L, IP injection, *P < 0.05), respectively. The Qn injected into the animals 30 min before sPLA_2_ was able to significantly reduce the myotoxic effect that was induced by the sPLA_2_ isolated from *Crotalus durissus terrificus*. Qn exhibited a neutralizing effect that was two times higher than the effect induced by Q.

## 4. Discussion

Quercetin (Q) is considered one of the most abundant natural flavonoids and it is mainly found in fruits and other foods. Quercetin is typically consumed in its glycosylated form as quercitrin (Qn), but multiple studies carried out with the aglycone form demonstrated its potent anti-inflammatory effect. However, the *in vivo* effectiveness of this compound has been questioned. The results of experiments on *in vivo* models of inflammation showed that Qn was more effective in reducing inflammation in comparison to Q, which showed better results in the *in vitro* assays [18, 28, 29].

Therefore, to shed some light on the inhibitory role of Qn in the inflammatory process, the effect of this flavonoid was evaluated in a typical sPLA_2_ purified from the venom of *C. d. terrificus* by using several experimental and theoretical methods, including chromatography, circular dichroism, molecular docking, and other *in vitro* and *in vivo* biological assays. Chromatography showed that binding to Q did not change the retention time of sPLA_2_ relative to sPLA_2_ : Qn samples, which exhibited a longer retention time than the native sPLA_2_ and sPLA_2_ : Q. This finding suggests that Qn may have caused structural changes in sPLA_2_, as also indicated by the results from circular dichroism and fluorescence scanning assays. These structural changes may be caused by the molecular interactions of Qn with the *C. d. terrificus* sPLA_2_, which could involve hydrogen bonding, hydrophobic and electrostatic interactions between Qn and the amino acid residues Gly 30, Gly 32, His 48, and Asp 49 and the Ca^2+^ ion as suggested by the molecular docking results. Indeed, crystal complexes of porcine pancreatic phospholipase A_2_/berberine (PDB ID 4DBK), *Daboia russelii pulchella* sPLA_2_/berberine (PDB ID 2QVD, [30]), and acidic *Bothrops jararacussu* sPLA_2_ (BthA-I)/p-bromophenacyl bromide presented similar ligand/protein interactions to those of the *C. d. terrificus* sPLA_2_/Qn docking complex, that is, involving amino acids from the Ca^2+^-binding loop (e.g., Gly 30) and catalytic site (e.g., Asp 49 and His 48).

The results of the enzyme kinetic studies show that the inhibition induced by quercitrin (Qn) is not the same as that observed for quercetin (Q), and this finding is apparent after 40 minutes (Figure 4(a)). This difference in the inhibitory capacity of Q and Qn against the sPLA_2_ from *Crotalus durissus terrificus* is supported by the results shown in Figure 4(b), which demonstrate the kinetic behavior of native sPLA_2_, of sPLA_2_ with quercetin, and sPLA_2_ with quercitrin. The difference between the flavonoids is most likely caused by the presence of a rhamnose sugar in Qn in accordance with the docking studies presented in Figure 3, which shows the insertion of Qn in the hydrophobic channels of sPLA_2_. Rhamnose appears to inhibit the substrate's access to the sPLA_2_ catalytic site.

An analysis of the enzymatic and pharmacological tests performed with sPLA_2_ and sPLA_2_ that were previously treated with both flavonoids strongly suggests that the enzymatic activity of sPLA_2_ is not crucial for edema or the myotoxic effects induced by sPLA_2_. The partial protein unfolding induced by Qn significantly contributes to the decrease in the edema and myonecrosis induced by native sPLA_2_ but did not abolish these effects. Pretreating sPLA_2_ with Qn induced more protein modifications than pretreating with Q. The changes in the pharmacological activity of the sPLA_2_ that was pretreated with Q or Qn indicate that the calcium loop region may be involved in the molecular interaction between the sPLA_2_ from *Crotalus durissus terrificus* and the receptors. In previous studies, Lambeau et al. used a Ca^2+^ loop mutant derived from sPLA_2_ that was isolated from venom to demonstrate the importance of this loop in the sPLA_2_ interaction with the M-type receptor [31].

sPLA_2_ from venom has been found to interact with a variety of mammalian sPLA_2_-binding proteins, such as N- and M-type receptors, 14-3-3 proteins and calmodulin, pentraxins and associated proteins, crocalbin, pulmonary surfactant proteins, KDR VEGF receptor 2, and factor Xa [32]. Furthermore, Rouault et al. also demonstrated that not only is the calcium binding loop region involved in binding to the receptor, but the interfacial binding domain is also involved. Thus, the stereochemical inhibition from when the substrate was binding to the active site of sPLA_2_ (as induced by Qn) could explain the different degrees of inhibition for quercetin (Q) and quercitrin (Qn).

Catalytically active sPLA_2_ can induce various biological and pathological effects, as in the sPLA_2_ present in snake venom. Generally, PLA_2_ causes these biological, physiological, and pathological activities through its enzymatic activity, which result in the increased production of arachidonic acid, which is the rate-limiting step in the generation of eicosanoids and platelet activating factors. This effect is caused by increased levels of intracellular arachidonic acid that stimulate the activity of cyclooxygenase 2 [33–35] and induce an increase in free radical peroxides and pro inflammatory cytokines. The increased levels of hydrogen peroxide may therefore lead to an increase in the lipid peroxidation levels, which can lead to cell membrane lesions such as those in skeletal muscle cells. Furthermore, sPLA_2_ can reportedly increase the mobilization of internal calcium through an indirect mechanism. This mobilization may lead to the activation of calpain, a member of a cytoplasmic protease family that can stimulate the activity of xanthine oxidase. This activity can lead to an increase in the concentration of molecular oxygen and may further exacerbate cellular injury [36, 37]. Several studies showed that Q and Qn are potent antioxidants. Results obtained by other authors demonstrated that the protective effect of these two flavonoids may be caused by their ability to neutralize the cytotoxic action of free radicals [38, 39]. The difference in the levels of protective or neutralizing effects observed between Q and Qn treatments may be caused by the presence of rhamnose because the only difference between Q and Qn is the presence of this sugar.

According to Lespade et al., [40], Kim et al., [41] glycosylation may increase the antioxidant properties of flavonoids [40, 41]. Moreover, Qn confers better protection than Q in some cases by protecting cells from ROS generation as well as ROS side effects [42]. The results in Figure 6 show that pretreating animals with Q and Qn can greatly reduce the toxic activity of sPLA_2_. These results also suggest that the action of these compounds occurs at the intracellular level and involves the neutralization of ROS and ROS side effects such as the activation and enhancement of the inflammation cascade. The presence of rhamnose in Qn is crucial to its protective activity against sPLA_2_ from *Crotalus durissus terrificus* in both *in vitro* and *in vivo* studies, which indicates that quercitrin (Qn) is more effective than quercetin (Q) at the cellular level. Qn inhibits the interfacial binding domain of the sPLA_2_ from *Crotalus durissus terrificus* from interacting with its receptor.
